# The Technique of Endovascular Intracranial Revascularization

**DOI:** 10.3389/fneur.2014.00246

**Published:** 2014-11-24

**Authors:** John J. Connors, Joan C. Wojak, Blaine H. Hoppe

**Affiliations:** ^1^Vanderbilt University Medical Center, Lafayette, LA, USA; ^2^Louisiana State University School of Medicine, Nashville, TN, USA; ^3^Our Lady of Lourdes Regional Medical Center, Lafayette, LA, USA

**Keywords:** intracranial stenosis, stroke, angioplasty, stenting, technique

## Abstract

Intracranial atherosclerosis was traditionally believed to carry a risk of stroke of 8% to 22% per annum. The annualized stroke rate in the recent stenting and aggressive medical management for preventing stroke in intracranial stenosis (SAMMPRIS) trial medical management arm was 12.2%. This trial was halted due to excessive periprocedural events in the stent arm. This stroke rate is still unacceptably, high and a treatment strategy is still needed. SAMMPRIS has no bearing on angioplasty alone. Angioplasty alone has always been our primary intervention for intracranial atherosclerosis and remains so to this day due to its relative simplicity, low complication rate, and efficacy. We have, however, made adjustments to our patient management regimen based on the results of SAMMPRIS. This paper outlines our current patient selection, procedural technique, and post-procedure management. The complications we have encountered while developing our technique are described along with how to avoid them and how to manage them. Our most recent results (since previous publications) are also discussed.

## Introduction

The results from the completed warfarin versus aspirin for symptomatic intracranial disease study (WASID) demonstrated that the rate of subsequent stroke was 18% in the first year for patients with symptomatic [transient ischemic attack (TIA) or stroke] stenoses of ≥70% on “best medical therapy” (1, 2). The annualized stroke rate in the medical arm of the recently published stenting and aggressive medical management for preventing stroke in intracranial stenosis (SAMMPRIS) was 12.2% (3, 4). This improvement from WASID was achieved by utilizing aggressive medical management that included dual antiplatelet therapy with intensive management of vascular risk factors combined with aggressive lifestyle modification (3, 5). Even so, the subsequent 12.2% stroke risk is still unacceptably high. Therefore, a treatment strategy is still needed.

The SAMMPRIS trial was prematurely stopped due to excessive periprocedural events in the stent arm. These results, however, have no bearing on the procedure of angioplasty alone and the associated risks and benefits, nor does SAMMPRIS have implications concerning the much simpler procedure utilizing balloon-mounted stents. The failed stenting of symptomatic atherosclerotic lesions in the vertebral or intracranial arteries (SSYLVIA) trial examined balloon expandable stents; all 30-day complications were in locations not recommended by the authors (6). The Vitesse intracranial stent study for ischemic therapy (VISSIT) trial also did not incorporate the patient selection described here but final results are not known; the trial was stopped prematurely (7).

Angioplasty has always been our primary intervention for intracranial atherosclerosis and remains such to this day due to its simplicity, low complication rate, and proven efficacy. Following SAMMPRIS, however, we adjusted our overall patient management regimen, as we will highlight below.

## Methods: Current Practice

### Patient selection

All patients presenting with transient ischemic attack or stroke undergo cross sectional imaging [computed tomography (CT) or magnetic resonance imaging (MRI)], with vascular studies [CT angiography (CTA), MR angiography (MRA)]. Patients with intracranial lesions thought to be the cause of the presenting event (>70% stenosis by North American symptomatic carotid endarterectomy trial (NASCET) criteria or <1 mm residual lumen in the artery supplying the affected territory) are placed on maximal medical therapy as utilized in SAMMPRIS (3, 5). This is managed by the neurologist (and cardiologist if the patient is under the care of one) and is adjusted as needed to address each patient’s specific condition and risk factors. Only patients who continue to have symptoms on maximal medical therapy are considered for endovascular therapy. Patients with incidentally discovered asymptomatic lesions are considered for endovascular therapy only if they have a high-grade stenosis (>70% and/or <1 mm residual lumen) and require major surgery such as coronary artery bypass grafting.

### Rationale for technique

Our technique and its rationale evolved over the first decade of our experience (~1990–1999) and have been described (8). A primary goal in the development of any procedure is to minimize periprocedural complications. In this instance, this was further driven by the belief that complications could outweigh any minor benefit for stroke prevention, later confirmed by SAMMPRIS (4). Observations led to certain conclusions: (a) procedural simplicity fosters procedural success, (b) excessive or rapid stretching of the vessel leads to intimal damage (8), (c) selecting a balloon approaching the size of the vessel more frequently leads to dissection, (d) intimal damage can lead to acute or subacute thrombosis, occlusion and/or stroke (8–10), and (e) intimal dissection leads to recurrent stenosis (10). It was, and is, our belief that the diseased vessel lacks flexibility and is more fragile and brittle. This fact led us to the analogy that this diseased vessel is like old leather: tough and easily cracked. Very slow stretching seemed to reduce cracking.

Past attempts to achieve a nearly normal vascular channel resulted in complications. While excellent post-angioplasty images might be obtained, important perforators have been sheared off even without visible dissection. This problem has been observed not where plaque is located (where the wall is thick and tough) but rather in normal areas of the wall opposite the plaque (where the wall is thin). This result could possibly be due to various layers of the vessel wall (intima, media, etc.) stretching at differing rates or in different directions, thus, leading to occlusion of the trans-wall microvascular channels. “Snowplowing” of plaque into (or onto) a perforator is a serious consideration but is rare in our observations.

### Pre-procedural management

Diagnostic cerebral angiography is generally performed as a separate procedure in order to assess collateral flow and hemodynamics as well as to plan the intervention. It also allows a more accurate estimation and discussion of the risks and benefits of the procedure for that specific patient. Treatment options and timing are discussed with the patient and family. The potential use of “off-label” devices is explained. In general, patients continue to receive all medications until endovascular treatment except when contraindicated; the exception to this would be for the isolated posterior (vertebrobasilar) circulation. Stenoses in an isolated posterior circulation can cause secondary systemic hypertension in an effort to perfuse the brainstem. When normal perfusion is restored, the blood pressure can drop precipitously. We hold the blood pressure medications on the day of the procedure for patients undergoing treatment in an isolated posterior circulation. Reperfusion hypertension can lead to hemorrhage, however, and continuous close observation is required after reperfusion in case the blood pressure needs to be acutely lowered.

### Procedure

#### General

All procedures are performed under general anesthesia. It is difficult or impossible to perform the entire procedure with the necessary finesse while having even minor patient motion; eventually there will be a problem. The majority of procedures are performed via a common femoral artery approach. Distal vertebral and/or basilar artery angioplasties/stents are occasionally performed via an ipsilateral brachial artery approach if there is access difficulty from a femoral approach.

#### Guide catheter

The position and stability of the neurointerventional guide catheter is critical. Every effort should be made to place the tip as close as possible to the lesion in the intracranial portion of the internal carotid artery (ICA) (typically petrous), or the level of C1 or C2 in the vertebral artery. Lack of safe, stable, and adequately distal position can “make or break” the procedure. A distal position of the guide catheter will help ensure a stable platform for delivery of the microcatheter/balloon and facilitate navigating the lesion with the necessary finesse. Once the working catheter and microguidewire are outside the guide catheter, they will take the outside track in all curves, thus, displacing the vital vectors of force. Once vectors are displaced, all friction exponentially increases and the “memory” in the shaft of the microguidewire can produce stored energy while torquing or steering. Distal microguidewire and catheter manipulation no longer have finesse and are more unpredictable. This distal guide catheter position will also simplify delivery of a stent if needed.

Modern guide catheters have significantly improved our ability to position the tip where necessary [e.g., Neuron (Penumbra, Inc., Alameda, CA, USA)]. Sometimes, however, a high position of the guide catheter is not enough to ensure its stability due to markedly tortuous proximal vessels. In these patients we advise the use of tri-axial system [e.g., a Shuttle sheath (Cook Inc., Bloomington, IN, USA)] through which a neurointerventional guide catheter is placed intracranially (e.g., Neuron). Indeed, a tri-axial system is now frequently employed (see Distal Tri-Axial Guide Catheter).

##### Telescoping distal access technique

If a carotid or vertebral artery is particularly tortuous and catheterization with a standard wire is difficult or dangerous, we recommend performing a telescoping access maneuver. Place the guide catheter proximal to the difficult access and load a microcatheter and microguidewire through a Y-connector. Select the vessel with a microguidewire and navigate the tortuous curves, following with the microcatheter. Once the microcatheter is well downstream, remove the microguidewire and replace with a stiff microwire, either regular length or exchange length. If the guide catheter itself is made coaxial by the use of an inner distal access catheter [DAC (Stryker Neurovascular/Concentric Medical, Mountain View, CA, USA)], the stiffer exchange-length microwire can provide sufficient stability to allow advancement of the distal access catheter followed by the guide catheter. In this way, there is progressive straightening of the vessel and never a large wire or blunt face of a guide catheter snowplowing on the vessel and potentially causing a dissection.

##### Proximal tri-axial stabilized guide catheter

If there is a congenitally small vertebral artery, a sheath (e.g., 8 Fr Shuttle) can be positioned in the subclavian artery proximal to the vertebral artery origin. First select the axillary artery and position an exchange wire [e.g., Connors exchange wire (Cook Inc.,)] with the tip distally in the axillary artery. Follow with the Shuttle sheath into the subclavian artery and then replace the exchange wire with a stiff exchange-length microwire [Hi-Torque Sparatcore (Abbott Vascular, Abbott Park, IL, USA)] and secure this wire. The sheath can then be withdrawn to a point just proximal to the vertebral artery without fear of the guide catheter buckling into the aorta.

##### Distal tri-axial guide catheter

Many times it is necessary to provide stiffer support for the guide catheter. This can be performed by placing a long 8 Fr. sheath (e.g., Shuttle) into the carotid artery and then advancing the guide catheter through the sheath. For shorter patients, and depending upon where the tip of the guide catheter is to be placed, a 60–80 cm sheath [e.g., Raabe (Cook Inc.,)] is placed so that the guide catheter has adequate length distal to the sheath.

#### Intraprocedural and periprocedural medication

Oral dual antiplatelet medication is mandatory before all cases. With modern medications, this should never be an issue. Traditionally, aspirin (325 mg non-coated) and clopidogrel have been utilized, although newer antiplatelet agents are also utilized. Platelet inhibition is usually assayed when a patient fails medical therapy; it is increasingly assayed on a routine basis. When a patient is on a single antiplatelet agent prior to an elective procedure, the second agent is started and platelet inhibition is assayed prior to the procedure.

Intraprocedural anticoagulation (e.g., heparin, bivalirudin) is always administered. With the advent of modern antiplatelet medications, rarely is there a need for rescue with GP IIb/IIIa inhibitors such as abciximab. The exceptions to this rule include emergent procedures, such as angioplasty performed in the course of acute stroke therapy, where a GP IIb/IIIa inhibitor might be necessary for bridging until oral antiplatelets take effect. This is typically necessary for clopidogrel, but aspirin is effective within minutes when administered orally or rectally. Other newer antiplatelet agents (e.g., ticagrelor) are also more rapidly effective than clopidogrel.

When any GP IIb/IIIa inhibitor is used, a very dilute mixture is infused very slowly in order to bathe the thrombus over an extended period: minutes, not seconds. While a systemic dose can be infused rapidly (and thus recirculate to achieve benefit), a local intra-arterial dose is very effective without producing systemic effects when administered in this manner.

Any reperfused territory can be susceptible to reperfusion hemorrhage, but isolated vascular territories (those without circle of Willis collaterals) are particularly susceptible. For hypertensive emergencies, it is mandatory that intravenous (IV) labetalol bolus (not nicardipine drip) be immediately on-hand if needed. Labetalol (a mixed alpha/beta adrenergic blocker) is specifically intended for use in hypertensive crises (start with 10 mg IV bolus followed by 20 mg every 2–5 min up to 200 mg.). On the other hand, nicardipine (a calcium ion influx inhibitor/“slow channel” blocker or calcium channel blocker) is only intended for infusion and is safe but slow even as a bolus (effects take several minutes).

#### Choice of balloon

Angioplasty alone is almost always our intention. Occasionally we will choose primary stenting (see below). Secondary stenting is only performed in rare circumstances such as when the lesion does not respond to repeated angioplasty, there is unacceptable rebound stenosis (return to the pre-angioplasty degree of stenosis or worse), or there is a resultant large dissection.

Most balloons have coronary indications, as do the balloon expandable stents we primarily use. The shortest balloon that will comfortably cover the length of the stenotic segment is chosen, typically 9 or 10 mm. A longer balloon will typically straighten the vessel, thus, stretching the shorter length of the inner curvature and possibly causing a dissection. The balloon diameter is undersized relative to the vessel diameter by 0.25–0.5 mm as previously described (8–11). In most cases, an over-the-wire system is utilized due to superior tracking ability, more sensitive “push–pull,” and appreciably more accurate control of the microguidewire when compared with a rapid exchange (monorail) system.

With modern tools and utilizing first-pass over-the-wire balloon technique, crossing a lesion is rarely a problem. Rapid exchange balloon systems perform far worse than over-the-wire systems for this challenge. Due to the fact that the wire is external to the shaft of the catheter, it will follow a different path from the catheter. This results in the development of excessive friction as well as stored energy. The wire will be forced to reenter the catheter and the combination of these factors will impede subtle and accurate microguidewire tip movement. We strongly believe that over-the-wire systems are more suitable for distal intracranial work. A monorail system can be utilized in the setting of proximal lesions (e.g., petrous or cavernous segment of the ICA or distal vertebral artery) and relatively straight vessels.

#### Choice of stent

Multistep self-expanding stents are rarely used unless necessary to tack down a large dissection (a rare occurrence with the defined technique). This is due to the fact they these are indeed “multistep” and require perfect technique every time. Two pairs of hands are required, both of which need to work in concert. In addition, it may be very difficult to safely re-cross self-expanding stents.

We now make occasional exceptions and perform primary stenting for specific locations. These would include the bare segments of the distal vertebral artery, the pre-ophthalmic carotid artery or, rarely, the proximal basilar artery. For instance, a stent might be chosen for a large eccentric plaque in a large vessel (proximal basilar artery) or for a larger artery in a segment known to have no perforators (distal vertebral artery proximal to the posterior inferior cerebellar artery). Stents are always the shortest available (8 mm if possible) and, of course, undersized. Even so, these stents are frequently hanging free in the vessel at one end of the plaque or the other with no clinical implications (free portions of stents are frequently present in vertebral origin or carotid stenting).

#### Procedural technique

Immaculate preparation of the balloon is mandatory if you wish to actually see the balloon. When inflating a balloon, the first thing that actually enters the balloon will be air and this must be essentially 0. A fully inflated 2 mm × 10 mm balloon will hold <0.06 ml of fluid. If any appreciable amount of air is present, the procedural damage might be done before you even see the balloon. Last second preparation with instant vacuum occasionally results in almost no contrast whatsoever (all air) in these micro-balloons. Therefore, we prepare these tiny balloons before the procedure with repeated vacuum syringes/stopcocks and replacing all air with contrast before advancing into the brain. A short microtube (30 cm) or luerlock can remain in place attached to the balloon port that can then be used to inflate the balloon after it has reached final position.

With the recommended technique, true inability to access the lesion is now rarely a cause of procedural failure (our technical success rate over the past 7 years has been 100% in 121 patients). Utilizing modern devices, most cases are performed with a first intention direct approach with an over-the-wire balloon catheter and microguidewire [e.g., Transcend.014 EX Platinum (Stryker Neurovascular, Fremont, CA, USA)]. Accessing the lesion is a two-step process. The first step is simply getting the balloon to a point just proximal to the lesion. This is accomplished with task-specific roadmaps for aid in selecting the intracranial site just proximal to the stenosis. For instance, traversing the petrous and cavernous carotids requires different views and less magnification than those for the highly important part of actually traversing the middle cerebral artery stenosis for the first time. Once the proximal position has been reached, repositioning the image intensifiers will be necessary. One plane will be chosen with maximum magnification for the absolute best view of the stenosis itself (at right angles) with minimal bone overlap. The other plane is used for overall supervision of the procedure. The field of view must include a good view of the stenosis and an optimal view the targeted location for the distal tip of the microguidewire. Being able to see the distal tip of the microguidewire for the entire procedure is mandatory.

Certain lesions (carotid siphon, basilar artery) can be shelf-like and on the outer rim of a sharp turn. Simply traversing the stenotic region is *NOT* the goal. If the microguidewire crosses the stenosis *within the plaque*, balloon inflation will then displace the plaque into the vessel lumen and make the situation worse or critical. It is imperative to avoid dissection of the plaque when crossing it. Finesse, patience, a stable and distal guide catheter position and technical skill are all necessary to traverse particularly irregular or eccentric stenosis. For difficult lesion access, the use of a tight J-shaped curve may be helpful by keeping the microguidewire in the central lumen. The wire tip can safely choose its path better than the operator. If primary direct approach angioplasty is not possible safely, or if secondary stenting is to be performed, a low-profile microcatheter designed for intracranial use may be advanced across the stenosis over the microguidewire. The microguidewire is then replaced with a microexchange wire [e.g., X-Celerator (Covidien/ev3 Neurovascular, Irvine, CA, USA)] after placing a very tight (~2 mm) P-shaped curve at the tip of the exchange wire to more safely allow for the inevitable to-and-fro motion of the wire tip for the next 30 min (at least). The balloon or stent is advanced over this wire.

Experience has taught us that inflation needs to be extremely slow with a goal of about 1 min to reach 1 atm and 4 min for complete inflation. Self-control is very important; one way to accomplish this is to very slowly inflate the balloon to <1 atm and/or when the balloon can be *barely* seen, set the indeflator down, and walk away. Even when stenting is performed, minimizing underlying vessel wall damage is still the goal since intimal damage can cause secondary intimal hyperplasia with resultant restenosis (9, 10). Therefore, we still inflate very slowly but admittedly faster than with angioplasty.

#### Observation period

After angioplasty or stenting is performed, the balloon/delivery catheter is withdrawn proximal to the stenosis, *leaving the microguidewire (or microexchange wire if used) across the stenosis to preserve access*. Intraprocedural observation of the angioplastied/stented site is performed to observe for three possible sequelae: rebound stenosis, significant dissection, and/or acute/subacute thrombosis. With the use of pre-operative dual antiplatelets, the latter has been essentially non-existent as opposed to early experience when subacute appearance of clot was not infrequently observed. Follow-up angiography is performed twice within 20–30 min to ensure the absence any of the complications mentioned above; there is always delayed observation. If rebound stenosis is observed, prolonged repeat angioplasty with the same balloon (possibly to a higher pressure and longer interval) is initially performed. Be aware that this can then result in the very complication to be avoided: dissection. Be careful and judicious. Rarely, a larger balloon may be used or a stent might be tried, although it is best to avoid (a) catheter/balloon exchanges, and (b) use of a secondary stent (both thought to be primary causes of the periprocedural complications in SAMMPRIS). This is almost always possible. Even self-expanding stents are clumsy intravascular instruments in comparison to angioplasty alone. If a large dissection is observed with subsequent thrombus, abciximab may be used along with patient observation to avoid use of a stent. Even clearly visible severe dissections typically heal if adequate flow is maintained for an hour.

If occlusion or dissection progresses and rescue stenting becomes necessary, the necessity of maintaining distal microwire position is clear. If necessary, the working catheter/balloon can be advanced safely back through the lesion over the microguidewire and a microexchange wire can then be placed. The choice of a rescue stent (balloon mounted or self-expanding) depends greatly not only on the anatomy but also on personal skill and that of your team. Most of these problems occur in short vessel segments in curved arteries and necessitate a self-expanding stent but our recent experience indicates that the need for a stent or exchange technique is rare.

### Post-procedure management

A CT scan is obtained immediately after the procedure if there is angiographic concern, or any time that a change in neurological status raises suspicion of procedural complication.

The vascular territory downstream of the target lesion may not have seen systemic pulse pressure in quite some time. In order to prevent reperfusion/hyperperfusion hemorrhage strict attention is paid to blood pressure with the goal of keeping the systolic pressure within a specified low-normal range (110–140 mm Hg) utilizing IV labetolol or nicardipine drip. (A single reperfusion hemorrhage inspires a lifetime of caution). As previously discussed, in our experience the patients with the highest risk of reperfusion hemorrhage (or headache) are those who had very poor collaterals (pial or circle of Willis), resulting in an isolated territory. In this circumstance the vascular bed will be maximally dilated.

Patients are initially managed in the intensive care unit. Once stable, they are transferred to the stroke unit. Most patients are ready for hospital discharge (or transfer to rehabilitation in the case of patients presenting with an acute stroke) within 24 h of the procedure. Discharge medications are discussed with the attending neurologist who will be following the patient. All patients are discharged on dual antiplatelet therapy unless warfarin therapy is required for another condition such as atrial fibrillation in which case aspirin alone is added (81 mg/day).

### Follow-up

All patients are followed clinically 1–2 weeks after discharge. All patients are evaluated with cerebral angiography 8 weeks after the procedure. If the follow-up appearance is satisfactory, imaging is repeated at 3 months, 6 months, 1 year, and then at yearly intervals using CT or MR angiography. Dual antiplatelet therapy is continued at least until stability is demonstrated on the 3-month images. Any decision regarding a change to single antiplatelet therapy is made in conjunction with the neurologist (and cardiologist if applicable).

If a *significant restenosis* (>70%) is demonstrated at the time of follow-up, the patient may undergo repeat angioplasty (or stenting if necessary) even without recurrent symptoms (as per local protocol) and is then followed as described above. The rationale for treating asymptomatic restenoses is twofold. WASID confirmed that a certain percentage of patients will not have recurrent symptoms manifested by TIA but rather by stroke or death (1, 2) and these patients have already failed a trial of maximal medical therapy for this lesion. We have also learned that repeat angioplasty is extremely low risk with an event rate approaching 0 (none in a decade). Asymptomatic restenoses of lesser severity are observed with repeat angiography and only treated if they became symptomatic.

Whenever a patient experiences symptoms possibly related to the treated stenosis, an MRI is obtained (or a CT if necessary) and an angiogram is performed. Repeat angioplasty is performed if indicated. Rarely, a lesion related to atherosclerotic plaque is encountered that continues to develop significant restenosis despite multiple interventions. In our early experience, some progressively stenotic lesions were observed that responded poorly to primary and repeated angioplasty as well as to stenting. Indeed, these behaved similarly to moyamoya disease but were not at the ICA terminus. These stenoses were typically in a single location and were usually found in the middle cerebral artery or supraclinoid ICA. They had smooth gradually tapering edges not typical of atheromatous plaque. These are currently thought to represent a form of inflammatory obliterative vasculopathy, similar to moyamoya disease. We now identify these lesions in advance and do not intervene but rather use maximum medical therapy, which for us includes cilostazol (12). No matter the treatment, these have a poor natural history. Encephaloduroarteriosynangiosis (EDAS) can be performed if indicated.

## Complications, How to Avoid Them, and Their Management

As discussed previously in the rationale for technique, we observed early in our experience that excessive or rapid stretching of the vessel and use of a balloon approaching the diameter of the vessel in size lead to intimal damage; intimal damage can lead to acute or subacute thrombosis, occlusion and/or stroke (8–10), and intimal dissection leads to recurrent stenosis (10). The resultant changes in technique have significantly reduced the incidence of complications. In current practice, if slightly excessive micro-dissection is seen, it is usually associated with “hurried” inflation.

The complications associated with the phases of development of our technique have been reported previously (8, 9). In current practice, periprocedural complications (within 24 h) are rare and minor. Specifically, the 30-day event rate over the past 7 years has been 2.5% (3/121 procedures), all minor. One patient presented with a symptomatic recurrent stenosis 27 days after angioplasty. Another patient had a very resistant eccentric stenosis in the cavernous ICA that required inflation of the balloon to 8 atm pressure and had a resultant dissection, which was treated conservatively and was healed on the first follow-up angiogram. The third patient had a transient neurological deficit of unclear etiology that resolved within 24 h; MRI obtained at the time did not reveal any acute findings.

### Guide catheter problems

All guide catheter problems should be avoidable; if there is doubt as to sufficient positioning with sufficient support, withdraw, and attempt another day. A final evaluation of the parent vessel and guide catheter location should always be performed at the end of the case. Thrombus forming around or in the guide catheter is related to procedural technique. Simply observing distal flow during injection does not indicate flow around the body of the guide catheter itself; that is confirmed by watching run-off, not the contrast injection. The most serious problem, in-catheter thrombus, is related to lack of constant adequate flush (*or slight back-bleeding*) with resultant stagnant blood in the guide catheter lumen. The final follow-up run will inject thrombus and reveal this situation.

### Parent vessel damage

Parent vessel damage is caused by poor guide catheter tip positioning, poor choice of guide catheter, initial manipulation, or intraprocedural movement. Stable position in the petrous (or more distal) ICA or C-2 vertebral artery level is recommended but admittedly might be difficult. It is imperative to choose the best guide catheter for the particular case in order to obtain a stable platform. The catheter tip will always be on an outer wall even if it looks like it is not. A suitable tip location with adequate support prevents the guide catheter for being forced to withdraw proximally, which could necessitate re-advancement into a high-tension system. The tip should not be positioned in a curved segment of the vessel where it will always be forcefully on an outer curvature and could cause dissection related to movement produced by the patient’s heartbeat and/or respiration. If dissection is significant, stent placement may rarely be necessary. Dissections heal with the medical management the patient is already on. Unless emergent, we do not work through recently damaged intima associated with a stent but rather let it heal and return at another date.

If spasm of the parent vessel is detected, the guide catheter should be withdrawn to a more proximal (and comfortable) position in the vessel and simply observed. While injection of nitroglycerine might alleviate the vasospasm quickly (50 mcg/ml of normal saline up to 100–200 mcg.) this is at the cost of significant vasodilatation of the downstream capillary bed. Simply waiting usually suffices and is particularly true in the case of acute stroke.

### Vessel damage from the distal microwire tip

It is important to use a suitable microguidewire designed for intracranial use with a soft and safe tip (e.g., Transcend.014 EX Platinum). Damage from the microguidewire tip typically occurs during initial wire manipulation or during active treatment of the lesion (balloon inflation). The single most hazardous event in every case is inadvertent movement of the distal microguidewire (or microexchange wire) tip causing perforation. The distal tip of the microwire must remain within the fluoroscopic field of view at all times during the procedure in order to ensure that it does not veer into an unseen small side branch where it has no room to buckle, with resultant perforation. While small branch perforations can be tolerated occasionally, this might not be the case when a patient is on dual antiplatelets and full anticoagulation. If necessary, once the lesion is crossed with the balloon, the microguidewire can be withdrawn and a tight P-shaped curve placed at the tip before advancing it. If a microexchange wire is used, the same curve should be placed at the tip.

The microwire tip should be advanced well downstream from the tip of the balloon past the stenosis and carefully positioned in a relatively straight segment of a vessel. In the anterior circulation this might be the M-1 segment of the middle cerebral artery or the M-2 segment (the inferior angular branch). Never intentionally place the microwire tip in the anterior division of the middle cerebral artery. The “candelabra” branches of the anterior division make sharp 180° turns in a distance of millimeters from the M-1 bifurcation and the guidewire (no matter how soft) will easily perforate at the bend rather than make the 180° turn in 3 mm. It is mandatory to take the time to position the tip of the microwire correctly and in the location recommended. If necessary, you can withdraw the microwire and reshape the tip; another reason to use an over-the-wire system.

After angioplasty, it is very important to maintain distal microwire position and avoid recrossing the lesion, particularly after manipulation. No matter how adept the operator believes himself or herself to be, it is not possible to skillfully advance a microguidewire through the center of the lumen of a newly dilated vessel regardless of whether or not there is visible dissection. Trying to accomplish this can result in worsened vessel dissection, perforation, or occlusion. If distal microwire position is lost, it may be necessary to accept the angioplasty result and maximize efforts to prevent and treat thrombus rather than potentially worsen the situation. Prolonged observation is usually sufficient. Another attempt can be made several weeks later.

### Thrombus formation and delayed occlusion at the site

In the early days of this procedure, delayed stroke (hours) was thought to possibly be the result of delayed vasospasm. We now understand that delayed stroke is due to delayed thrombus formation with or without progressive dissection leading to vessel occlusion. This was formerly the most common potential complication of this procedure. Delayed observation is therefore mandatory.

Exposed endothelial matrix is very thrombogenic. A small amount of intimal damage is inevitable when performing angioplasty or stenting, but visible thrombus is rarely seen in present practice. This is almost certainly due to the consistent use of preprocedure dual antiplatelet therapy. Visible subacute platelet clumping can be treated with GP IIb/IIIa inhibitors (e.g., abciximab). A very dilute and slow intra-arterial infusion will be vastly more concentrated than any serum level and will bathe the thrombus for a prolonged period with better clinical results. For abciximab, 5–10 mg in 30 ml injected over 5–10 min is usually sufficient and will not give an appreciable systemic effect.

### Dissection at the site of angioplasty

A small amount of vessel damage might be unavoidable. However, the best solution for a true “macro” dissection (visible intimal flap) is *avoidance*. The techniques of *undersizing the balloon and extremely slow inflation* were developed to minimize this risk (9). The rate of significant dissection and subsequent restenosis has been very low (8).

If a hemodynamically significant dissection does occur, the lesion is accessible, and the microwire still has distal position, a stent can be placed as a last resort. With or without a stent, the current dual antiplatelet therapy and intraprocedural anticoagulation typically maintains patency in the vessel, allows the situation to stabilize, and usually is sufficient.

### Emboli

There are three principal causes of downstream emboli, all rare. First, the guide catheter can produce thrombus (inside or out). Second, thrombus that forms at the angioplasty site can migrate downstream. Third, thrombus or plaque can be dislodged from the original lesion. The use of dual antiplatelet therapy has greatly reduced the incidence of emboli from this source. Thrombus at the site of the original lesion is usually encountered in the setting of a patient with recurrent or crescendo symptoms, presumably due to unstable plaque, and who fails medical therapy rapidly. If there appears to be thrombus present and it is necessary to continue with the procedure, it is better to treat thrombus *in situ* rather than downstream. Be patient and treat as described above.

Treatment of an embolus is dependent upon its composition. Acute thrombus is typically composed almost exclusively of platelets and responds well to GP IIb/IIIa inhibitors. The embolus will be relatively small and should migrate to a second- or third-order vessel; the time for rescue can be prolonged in this setting. If contrast reaches the thrombus, the therapeutic agent will also. Clinically significant emboli are rare in recent practice.

### Rebound stenosis

Rebound stenosis is the major drawback of angioplasty with an undersized balloon but this is not a complication. Purposely undersizing the balloon will prevent major intimal damage as completely as possible. This process will, however, cause some lesions to be inadequately dilated. A certain amount of damage may be necessary to produce sufficient dilation, but an inadequate result that must be retreated at a later date is preferable to a damaged vessel leading to intimal flap or occlusion. Recurrent stenosis secondary to intimal hyperplasia associated with healing of the angioplasty site is the reason for early evaluation at 8 weeks after angioplasty or stenting. In our experience, recurrent stenosis occurs very early in the post-procedure period.

Intrinsic elastic recoil does not appear to be as great a problem in intracranial lesions as in extracranial lesions such as the vertebral artery origin. Vessel geometry, however, can be a problem. The bend of a vessel (e.g., the curve of the distal vertebral artery at the skull base) is a poor location for effective angioplasty, and prone to kinking after angioplasty. Stent placement in these locations can also be problematic, but often produces a better result. This is a typical location for primary balloon-mounted stent use.

## Discussion

Clinical results have shown that modest angioplasty results produce satisfactory clinical results (8–11, 13, 14). A review of our experience over the last 7 years reveals that 121 procedures were performed. As noted above, there were three complications. Primary stenting was performed in 11 instances and secondary stenting in 3. There was 100% technical success. There has only been one patient who presented with a symptomatic recurrence, treated successfully. We attribute these results mainly to the decrease in significant dissection following angioplasty. Dissection has been shown prospectively to be a statistically significant predictor of not only stroke in the periprocedural period but also of restenosis at the follow-up angiogram (10). These facts confirmed our observational impression and affirm our mantra of “avoiding dissection at all costs.”

The complication rate of angioplasty alone in these different papers consistently was <4.5% for major and minor complication combined at 1-year post intervention. This complication rate for intracranial angioplasty provides a reasonable treatment strategy for symptomatic intracranial atherosclerotic stenosis with its attendant risk of at least 12.2% stroke/death within the first year (1–4).

Concerning the issue of intracranial stenting in treatment of symptomatic intracranial atherosclerotic disease (ICAD), we believe that the available self-expanding stent technology has been proved to be difficult to place without complications. Conversely, we have found balloon-mounted stents to be simple to use, safe, and effective when used in the recommended locations. We believe that stenting, both self-expanding and balloon expandable, should be reserved for particular situations.

In summary, experience has taught us much about the safe and efficacious performance of intracranial revascularization. The key points of our current technique, as discussed above, are as follows:

1. Careful selection of the guide catheter combined with patient, skillful placement of the tip in the recommended distal location is extremely important both for technical success and avoidance of complications.
2. The shortest balloon possible should be chosen and the diameter should be downsized relative to the vessel diameter.
3. Impeccable attention to detail with wire manipulation and crossing of the stenosis is mandatory. Distal wire tip positioning is crucial. The distal tip of the microwire is the greatest cause of periprocedural complications.
4. Excruciatingly slow balloon inflation is second only to submaximal sizing in importance for preventing dissection.
5. Secondary stenting should be avoided if possible.

## Conclusion

Although aggressive medical management has decreased the incidence of stroke associated with intracranial atherosclerotic stenosis, there is still a failure rate of >12% at 1 year. An important role for endovascular intervention remains. Angioplasty alone in carefully selected patients has repeatedly been shown to be technically feasible. Dissection should and can be avoided with the recommended technique. Studies have consistently demonstrated angioplasty alone to provide clinical benefit for intracranial atherostenosis better than stenting or medical management alone.

## Conflict of Interest Statement

The authors declare that the research was conducted in the absence of any commercial or financial relationships that could be construed as a potential conflict of interest.
